# Curve Appeal: Exploring Individual Differences in Preference for Curved Versus Angular Objects

**DOI:** 10.1177/2041669517693023

**Published:** 2017-04-03

**Authors:** Katherine N. Cotter, Paul J. Silvia, Marco Bertamini, Letizia Palumbo, Oshin Vartanian

**Affiliations:** Department of Psychology, University of North Carolina at Greensboro, NC, USA; University of Liverpool, UK; Liverpool Hope University, UK; University of Toronto, ON, Canada

**Keywords:** angularity, curvature, aesthetics, openness to experience, art expertise

## Abstract

A preference for smooth curvature, as opposed to angularity, is a well-established finding for lines, two-dimensional shapes, and complex objects, but little is known about individual differences. We used two-dimensional black-and-white shapes—randomly generated irregular polygons, and arrays of circles and hexagons—and measured many individual differences, including artistic expertise, personality, and cognitive style. As expected, people preferred curved over angular stimuli, and people’s degree of curvature preference correlated across the two sets of shapes. Multilevel models showed varying patterns of interaction between shape and individual differences. For the irregular polygons, people higher in artistic expertise or openness to experience showed a greater preference for curvature. This pattern was not evident for the arrays of circles and hexagons. We discuss the results in relation to the nature of the stimuli, and we conclude that individual differences do play a role in moderating the preference for smooth curvature.

## Introduction

People show a small but consistent preference for the round over the sharp, for the curvy over the pointy, when judging lines, shapes, abstract objects, and artifacts. This overall preference for curvature is now firmly established (Gómez-Puerto, Munar, & Nadal, 2015; for its historical context, see Bertamini & Palumbo, 2015) but not well understood. In the present research, we sought to illuminate this effect by examining individual differences in curvature preferences. Whether people differ in their preferences for curvature has been explored in only a handful of studies (e.g., Silvia & Barona, 2009), and the findings so far are inconsistent. Examining the role of individual differences in these preferences will thus extend research to date and inform the broader question of why people prefer curved over angular objects.

## The Robustness of Curvature Preferences

The notion that curved lines and objects are more pleasing has a long history in writings on aesthetics (e.g., Hogarth, 1753) and in empirical research. In early studies, curved lines were seen as quiet and lazy (Lundholm, 1921; Poffenberger & Barrows, 1924) or graceful and playful (Hevner, 1935), while angular lines were seen as agitating (Lundholm, 1921; Poffenberger & Barrows, 1924), harsh (Poffenberger & Barrows, 1924), or dignified (Hevner, 1935). Similar patterns were found when fonts were adjusted to be more rounded or angular (Kastl & Child, 1968).

During the past decade, researchers have revisited this issue of angularity and preference and have consistently found that people prefer curved things. This holds true for lines (Bertamini, Palumbo, Gheorges, & Galatsidas, 2016; Salgado-Montejo, Tapia Leon, Elliot, Salgado, & Spence, 2015), abstract novel shapes (Bar & Neta, 2006; Bertamini et al., 2016; Silvia & Barona, 2009; Velasco et al., 2016), common objects (Bar & Neta, 2006; Westerman et al., 2012), car interiors (Leder & Carbon, 2005), and interior architectural environments (Dazkir & Read, 2012; van Oel & van den Berkhof, 2013; Vartanian et al., 2013). Even infants (Jadva, Hines, & Golombok, 2010; Quinn, Brown, & Streppa, 1997) and great apes (Munar, Gómez-Puerto, Call, & Nadal, 2015) demonstrate this preference. The effect appears to stem from an appealing quality of curved things that motivates approach (Bertamini et al., 2016; Palumbo, Ruta, & Bertamini, 2015) instead of a threatening quality of angular, sharp things (Bar & Neta, 2006, 2007) that motivates avoidance.

Despite consistent evidence pointing to a preference for smooth curvature, however, the history of art suggests that preference for shapes is a complex phenomenon. Hogarth (1753), in his treatise on beauty, pronounced a wavy line as the *line of beauty*, but culture and artistic canons seem to matter as well. For instance, *abstract plasticism* is a style associated with Piet Mondrian (1872–1944) and characterized by simple straight lines and a complete absence of curves. This can be contrasted with *abstract expressionism*, associated with Jackson Pollock (1912–1956). In Pollock’s case, the paintings include splatters of paints that have an organic aspect and therefore include smooth curves reminiscent of those present in the natural environment (Taylor, Micolich, & Jonas, 1999; Taylor, Spehar, Van Donkelaar, & Hagerhall, 2011).

## What Moderates Curvature Preferences?

### Aspects of Stimuli and Tasks

On the one hand, the preference for curvature is solid and consistent, but on the other hand, it is necessary to study its boundary conditions, moderators, and mechanisms. Several within-person factors—aspects of the stimuli themselves—affect these preferences. For example, when semantic knowledge of the objects is relevant—such as curvy bombs and pointy teddy bears (Leder, Tinio, & Bar, 2011)—it can wash out the effects of low-level features like angularity. Likewise, cultural trends in product design can shape the background against which objects are evaluated (Carbon, 2010).

One factor worth examining is the kind of emotional response measured in studies of curvature. To date, the overwhelming focus has been on *preference*: The positively valenced appeal of curvy things, measured with self-report items associated with *pleasing*, *likeable*, and *positive* and with behavioral measures of choice and approach. Aesthetics research, for good reason, focuses heavily on positive feelings of liking and preference (Silvia, 2012). Nevertheless, pleasure and interest are distinct responses, and many pleasing things are not interesting (Graf & Landwehr, 2015). Many studies, from Berlyne’s (1971) early work to the present (Marković, 2012; Silvia & Kashdan, in press), show that liking and interest respond differently to low-level features like complexity and familiarity. It is possible that angular objects are less pleasing but more interesting, consistent with greater interest in objects that are complex, variable, and heterogeneous (Silvia, 2006).

### Aspects of People

At the between-person level—factors that differ between participants—little is known about variables that affect curvature preferences. Thus far, expertise in the arts has received the most attention. Compared with novices, people with training in the visual arts see, think about, and appreciate art differently (Parsons, 1987; Smith & Smith, 2006). In particular, a common finding is that experts are less affected by low-level, superficial features of a work (e.g., whether it depicts objects and colors people like) and more sensitive to compositional and historical features (e.g., Cleeremans, Ginsburgh, Klein, & Noury, 2016; Locher, 1996; Lundy, 2010; Parsons, 1987; Silvia, 2013). Because they think about art in deeper, more complex ways, art experts might be less affected by low-level visual features that appeal to novices. In two studies, Silvia and Barona (2009) found puzzling effects of expertise. In one study, novices showed stronger curvature preferences; in another, experts showed stronger curvature preferences.

Individual differences beyond expertise have not been explored. An obvious candidate is *openness to experience*, a trait that pops up throughout the psychology of aesthetics and creativity (Oleynick et al., 2017). People high in openness to experience are more engaged with the arts: They are more likely to find the arts interesting and valuable, have much more knowledge about art, and have formal or informal training in the fine and performing arts (McCrae, 1996; Silvia, 2007). More generally, people high in openness to experience are more imaginative, creative, unconventional, and sensitive to subtle emotions (Kaufman, 2013; McCrae & Sutin, 2009). Unlike art expertise, which develops over many years and is positively skewed, openness to experience is apparent early in life. Personality traits like openness to experience do develop, of course, but they are considered universal features of people (Nettle, 2009) rather than domains of acquired knowledge that some people choose to pursue.

## The Present Research

The present research delves deeper into preference for smooth curvature by exploring moderators, with an emphasis on individual differences. Our primary aim was to examine whether some people have stronger curvature preferences and to characterize the traits associated with such preferences. Variation in curvature preferences is an intriguing issue that, ideally, can inform theorizing about the basic mechanisms behind the preferences.

In the present study, a sample of young adults viewed and rated a set of randomly generated irregular polygons that varied in complexity (Bertamini et al., 2016). In addition, they also viewed and rated a set of arrays containing circles and hexagons that varied in degree of balance within a frame (Silvia & Barona, 2009). This second set was used to replicate past work and to see if curvature preferences were consistent across the sets. If preference for curvature is partly due to stable between-person factors, they should be consistent: People’s degree of curvature preferences should correlate across the tasks. Finally, we collected ratings of both liking and interest and measured viewing times to see if curved objects are more pleasing but less interesting and if curvature predicts how long people viewed an image. We expected, based on past work, to find an overall increase in liking for curved objects.

We included a wide range of measures of individual differences. Our approach was guided by past research on important factors in aesthetic judgment and preference. We focused on three classes of factors. First, we measured art expertise, a major moderator of aesthetic preferences and processes that had inconsistent effects on curvature preference in past work (Silvia & Barona, 2009). Second, we measured openness to experience, another major trait involved in people’s engagement with the arts and aesthetic preferences. We measured openness to experience with a cluster of scales that emphasize its different aspects (Oleynick et al., 2017). Finally, we measured a cluster of traits associated with systematic or deliberate versus heuristic or intuitive processing styles, such as need for cognition (Cacioppo, Petty, & Feng, 1984) and types of intuitive thinking (Pretz et al., 2014). This family of traits has not been widely studied in arts and aesthetics research, so these scales were included for essentially exploratory reasons.

## Method

### Participants

Participants were 132 undergraduate students enrolled in psychology classes who volunteered as part of a class research participation option. Thirteen people were excluded from the analyses due to exceeding the cutoff for inattentive responses (more than two) on an infrequency scale (see Maniaci & Rogge, 2014; McKibben & Silvia, in press, 2016). This resulted in a final sample size of 119 people, which is the basis of the following analyses. The final sample of 119 was primarily female (61%) and diverse in self-reported race and ethnicity (people could select more than one category): European American (48%), African-American (41%), Asian-American (10%), Hispanic (5%), and Native American (5%). On average, participants were 18.92 years old (*SD* = 1.28). The university has popular programs related to the fine and performing arts, and 12% of the participants had majors or minors in some area of the arts (primarily fine art, art history, dance, theatre, and music; see Silvia & Nusbaum, 2012).

### Procedure

Participants completed the study in small groups in a lab setting. Once participants provided informed consent, the researcher explained the broad purpose of the study. Participants then began the experiment, which was administered on individual computers using MediaLab. All participants viewed and rated the irregular polygons at the start of the study, followed by the arrays of circles and hexagons. All measures of individual differences were taken afterward. The study took around 45 minutes to complete.

### Materials

#### Irregular polygons

**Figure 1.**
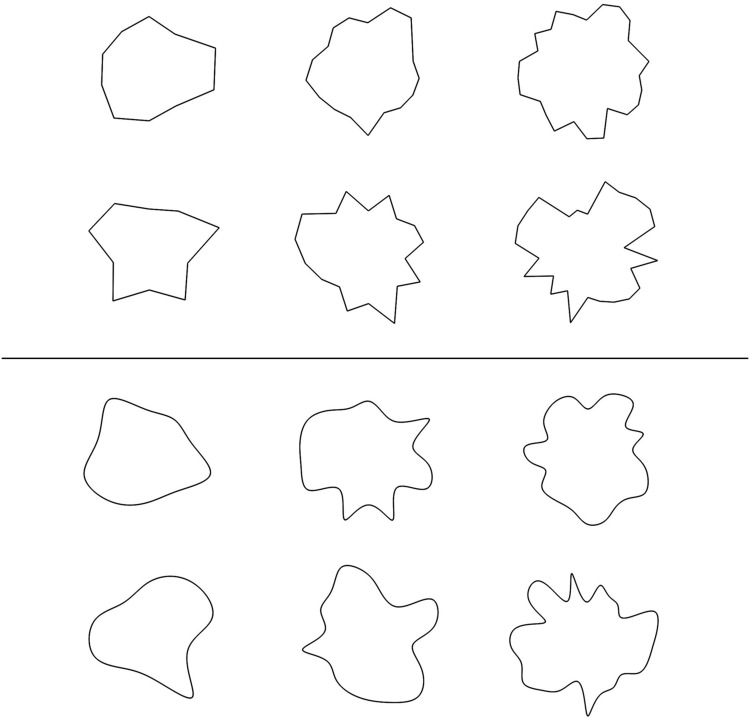
Examples of angular (top panel) and curved (bottom panel) irregular polygons. The polygons vary in the number of sides (three levels) and in their range (two levels).

Our stimulus set was a subset of randomly generated polygons created by Bertamini et al. (2016) and Palumbo and Bertamini (2016). By design they had some degree of complexity (so that they differed visibly one from the other) but they did not resemble any familiar object, nor were they composed of simple geometric parts (i.e., they could not be described, for instance, as two circles or a square and a hexagon). Figure 1 shows examples, and the full set is available on Open Science Framework (https://osf.io/vrjyp/). The stimuli were created by selecting locations along a circle, and by varying the length of the radius (between a minimum and a maximum value). This process created polygons with 10, 18, or 26 vertices. Next, polygons were altered to generate curved variants. To do so, a smooth curve was created by using a cubic spline applied to the original polygon. Complexity was also varied by using two values of the range of the radius used (i.e., the difference between the minimum and the maximum radius values). There were two levels of range, and they produced a difference in the amplitude of the indentations. The combination of factors (number of vertices = 3, range = 2, curvature = 2) gives 12 types of shapes. We used two sets for a total of 24 images. The images appeared in a random order. The participants could view each image for as long as they wished, but the images stayed on screen for at least 2 seconds. People clicked the mouse or space bar to advance to the ratings, and the viewing time was recorded by the software.

After each image, participants gave ratings of *pleasantness* (How PLEASANT is this image? 1 = *Not at all pleasant*, 7 = *Very pleasant*), *interest* (How INTERESTING is this image? 1 = *Not at all interesting*, 7 = *Very interesting*), and *complexity* (How COMPLEX is this image? 1 = *Not at all complex*, 7 = *Very complex*). The ratings appeared in the same fixed order for all items and participants.

#### Preference for Balance Test

**Figure 2.**
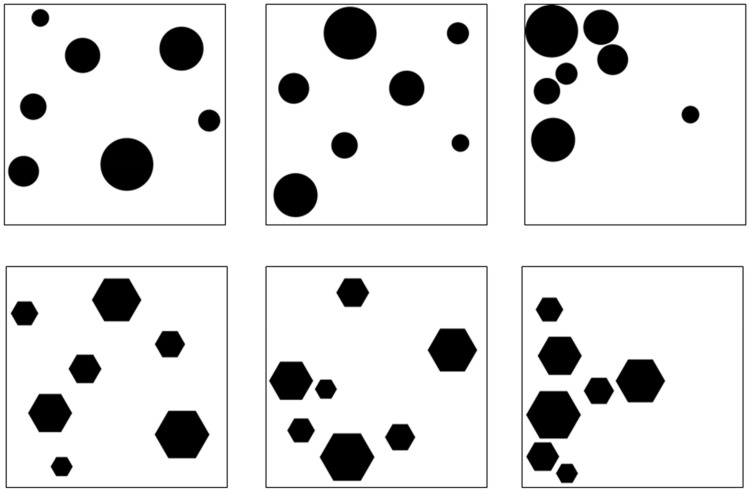
Examples of stimuli from the Preference for Balance Test. The circle (top) and hexagon (bottom) stimuli had three levels of imbalance: low (left), medium (middle), and high imbalance (right).

The Preference for Balance Test (Wilson & Chatterjee, 2005) was included to attempt to replicate past research (Silvia & Barona, 2009) and to evaluate if curvature preferences were consistent across different kinds of images. The Preference for Balance Test consists of arrays of circles (curved) and hexagons (angular). Eighteen images were presented: nine containing circles and nine containing hexagons. For each set of nine images, there were three images for each of three levels of imbalance (low, medium, and high). Sample stimuli are shown in Figure 2; all 18 images are available at Open Science Framework (https://osf.io/vrjyp/). This set was used in our past research (Silvia & Barona, 2009, Study 1). The images appeared in a random order. People clicked the mouse or space bar to advance to the ratings, and the viewing time was recorded by the software.

After each image, participants gave ratings of *pleasantness* (How PLEASANT is this image? 1 = *Not at all pleasant*, 7 = *Very pleasant*) and *interest* (How INTERESTING is this image? 1 = *Not at all interesting*, 7 = *Very interesting*). The ratings appeared in the same fixed order for all items and participants.

### Measures of Individual Differences

#### Aesthetic fluency scale

This scale was used to assess artistic expertise (Smith & Smith, 2006). Ten people or ideas from art history (e.g., impressionism and fauvism) were presented, and the participants rated how familiar they were with each one on a scale from 0 (*I have never heard of this artist or term*) to 4 (*I can talk intelligently about this artist or idea in art*). This scale has been used in many past studies as a brief measure of people’s knowledge of the arts (e.g., Silvia, 2007, 2013; Swami, 2013).

#### HEXACO 100 Personality Inventory–Revised

This inventory consisted of 100 items measuring six domains of personality: Honesty-Humility, Emotionality (Neuroticism), eXtraversion, Agreeableness, Conscientiousness, and Openness to Experience (Lee & Ashton, 2004). Each item was rated on a scale of 1 (*Strongly Agree*) to 5 (*Strongly Disagree*). We were primarily interested in openness to experience and its facets—*aesthetic appreciation*, *inquisitiveness*, *creativity*, and *unconventionality*—so only these scales were included in the analyses.

#### NEO-Five-Factor Inventory

This 60-item scale measures five domains of personality: Neuroticism, Extraversion, Openness to Experience, Agreeableness, and Conscientiousness (Costa & McCrae, 1992). Each item was rated on a scale from 1 (*Strongly Agree*) to 5 (*Strongly Disagree*). As our interest was in openness to experience, we examined only this domain in our analyses.

#### Big Five Aspects Scale

We used the Openness or Intellect subscale of the Big Five Aspects Scale (BFAS; DeYoung, Quilty, & Peterson, 2007). This 20-item subscale measures two aspects of openness to experience: *Openness* and *Intellect*. Each item is rated on a scale from 1 (*Strongly Disagree*) to 5 (*Strongly Agree*).

#### Need for Cognition

This 18-item questionnaire was used to assess how much people engage in and enjoy thinking (Cacioppo et al., 1984). Each statement was rated on a scale from 1 (*Strongly Disagree*) to 5 (*Strongly Agree*). Sample items include “I only think as hard as I have to” and “I feel relief rather than satisfaction after completing a task that required a lot of mental effort.”

#### Types of Intuition Scale

This scale was used to examine the ways in which people make decisions and solve problems (Pretz et al., 2014). Twenty-three statements (e.g., “I would rather think in terms of theories than facts”; “There is a logical justification for most of my intuitive judgments”) were rated on a scale from 1 (*Definitely False*) to 5 (*Definitely True*). These statements are grouped into four subscales: *Holistic Abstract* (thinking about a problem in abstract terms), *Holistic Big Picture* (focusing on the entire problem rather than details of the situation), *Inferential* (making decisions based on automatic, analytic processes), and *Affective* (making decisions by relying on emotional reactions to a situation).

## Results

### Multilevel Analysis

The design had both within-person factors (e.g., curved vs. angular images) as well as between-person factors (e.g., art expertise and openness to experience; see Table 1). The data were thus analyzed with multilevel models, which can flexibly model such designs. The analyses were run with maximum likelihood and robust standard errors in Mplus 7.4. The slopes and intercepts were random.

In this design, individual differences were factors that varied between people (Level 2), and aspects of the images (their curvature, complexity, and imbalance) were factors that varied within people (Level 1). For the irregular polygons, curvature was coded as 0 (angular) and 1 (curved), number of sides was coded as 0 (low), 1 (medium), and 2 (high), and range was coded as 0 (low) or 1 (high). Similarly, for the preference for balance arrays, curvature was coded as 0 (hexagons) and 1 (circles), and imbalance was coded as 0 (low), 1 (medium), and 2 (high imbalance). The Level 2 predictors (e.g., scales measuring art expertise and personality traits) were standardized, which centers them at 0 (the sample mean) and places them on the same standard deviation scale. Unless noted otherwise, all the regression coefficients are unstandardized. The intraclass correlations (ICCs) showed that around 40% of the variance in ratings of pleasantness (ICCs = .40 for the polygons, .47 for the circle or hexagon arrays) and interest (ICCs = .39 for the polygons, .42 for the circle or hexagon arrays) was at Level 2.

### Did People Prefer Curved Objects?

**Figure 3.**
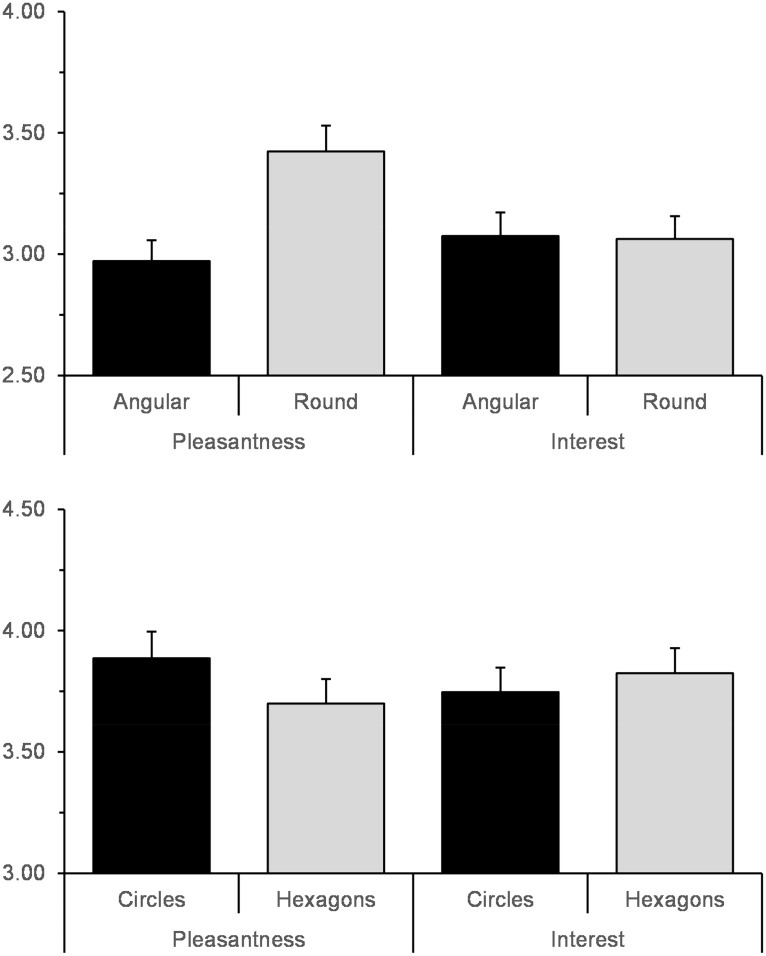
Main effects of curvature on pleasantness and interest ratings for the irregular polygons (top) and circles and hexagons (bottom). Error bars represent standard errors.

Based on past work, we expected that participants would prefer the curved objects more than the angular ones (see Figure 3). For the irregular polygons, participants reported liking the curved polygons more than the angular ones (*b* = .27, *SE* = .10, *p* = .005). Additionally, people liked polygons with more sides (*b* = .11, *SE* = .05, *p* = .036), which replicates the classic effect of complexity on preference (e.g., Berlyne, 1971).^1^

For the imbalanced arrays of circles and hexagons, people again showed a preference for curvature. Ratings of pleasantness were higher for the arrays of curved circles than the angular hexagons (*b* = .19, *SE* = .10, *p* = .070), although the effect was weaker than for the irregular polygons. Neither the level of imbalance nor the interaction between curvature and imbalance significantly predicted liking.

### Was Curvature Preference Consistent?

There was an overall preference for curvature for both irregular polygons and arrays of simple circles and hexagons. To see if there were individual differences in the tendency to prefer curved forms, we estimated the correlation between curvature preference for the irregular polygons and for the imbalanced arrays of circles and hexagons. For each set, a difference score was computed that reflected the difference in liking between the curved and the angular images. The correlation between curvature preference for the irregular polygons and for the circle and hexagon arrays was significant (*r* = .32 [.15, .50], *p* < .001) and medium in effect size (Cumming, 2012).

It thus appears that there were consistent between-person differences in curvature preference: People who preferred curved objects in one stimulus set were likely to prefer the curved objects in the other stimulus set.

### What Moderated Preference for Curvature?

The remaining question is to explore predictors of these between-person differences in preferences for curvature. We thus conducted multilevel models that examined between-person factors, such as personality traits and art expertise, to see if some factors predicted stronger curvature preferences. For simplicity, the irregular polygons and circle-hexagon arrays were analyzed separately, and only the main effect of curvature was included in the model (i.e., imbalance, complexity, and range were omitted). Note that because the between-person predictors are standardized, the coefficients represent the predicted change in the difference between curved and angular images for each 1 *SD* change in the predictor.

#### Irregular polygons

The first column of Table 2 shows the moderating effects of the between-person variables on curvature preferences. Some of the traits did significantly moderate preference. First, art expertise significantly enhanced the curvature effect: People who had higher scores on the aesthetic fluency scale showed a stronger preference for the curved over the angular polygons (*b* = .21, *SE* = .09, *p* = .021). Second, several of the scales measuring openness to experience showed similar effects. Curvature preferences were significantly greater for people scoring higher on the NEO openness to experience scale (*b* = .25, *SE* = .08, *p* = .002) and the HEXACO unconventionality facet (*b* = .41, *SE* = .10, *p* < .001); the increase was marginal for the overall HEXACO Openness to Experience scale (*b* = .16, *SE* = .09, *p* = .083) and the BFAS Openness scale (*b* = .15, *SE* = .08, *p* = .077). Finally, the Holistic Abstract subscale of the Types of Intuition Scale significantly moderated curvature preference: People who like thinking in abstract terms preferred the curved polygons more (*b* = .21, *SE* = .08, *p* = .008). None of the other individual differences we investigated was significant (see Table 2). Overall, then, a consistent profile appeared for the irregular polygons.

**Table 1.** Descriptive Statistics for the Individual Differences Variables (*n* = 119).

Variable	*M*	*Mdn*	*SD*	Min., Max.
Art expertise	1.79	1.70	.54	1.00, 3.90
HEXACO openness to experience	3.07	3.13	.58	1.69, 4.38
HEXACO O: Aesthetic appreciation	3.10	3.00	.86	1.25, 5.00
HEXACO O: Inquisitiveness	2.62	2.50	.79	1.00, 5.00
HEXACO O: Creativity	3.30	3.25	.82	1.00, 5.00
HEXACO O: Unconventionality	3.27	3.25	.48	2.00, 4.75
NEO openness to experience	3.55	3.50	.48	2.50, 4.92
BFAS: Openness	3.54	3.50	.52	2.20, 4.90
BFAS: Intellect	3.37	3.30	.55	1.70, 4.90
Need for cognition	3.04	3.00	.48	1.94, 4.33
TIntS: Holistic abstract	2.73	2.67	.68	1.33, 5.00
TIntS: Holistic Big picture	3.44	3.50	.60	1.75, 5.00
TIntS: Inferential	3.84	3.88	.43	2.63, 4.88
TIntS: Affective	3.23	3.25	.61	1.50, 5.00

BFAS = Big Five Aspects Scale; TIntS = Types of Intuition Scale.

**Table 2.** Preference for Curvature Moderated by Individual Differences.

	Irregular polygons			Circles and hexagons		
	*b*	*SE*	*p*	*b*	*SE*	*p*
Art expertise	.21	.09	.021	−.02	.08	.788
HEXACO openness to experience	.16	.09	.083	−.07	.09	.473
HEXACO O: Aesthetic appreciation	−.10	.10	.277	−.02	.05	.882
HEXACO O: Inquisitiveness	.12	.13	.246	−.15	.10	.131
HEXACO O: Creativity	−.10	.11	.335	−.03	.13	.803
HEXACO O: Unconventionality	.41	.10	< .001	.17	.12	.151
NEO Openness to experience	.25	.08	.002	.00	.08	.970
BFAS: Openness	.15	.08	.077	.07	.08	.384
BFAS: Intellect	.02	.08	.828	−.15	.08	.040
Need for cognition	.09	.07	.212	−.17	.09	.068
TIntS: Holistic abstract	.21	.08	.008	−.06	.07	.354
TIntS: Holistic Big picture	.05	.08	.558	−.05	.09	.542
TIntS: Inferential	.09	.08	.278	−.05	.08	.530
TIntS: Affective	−.01	.09	.886	.10	.10	.291

*Note.* BFAS = Big Five Aspects Scale; TIntS = Types of Intuition Scale. The *b* values are unstandardized regression weights. Because each trait is standardized, the coefficients for the same outcome are comparable to each other: They are the predicted change in the difference between curved and angular images (in raw scale units) for each 1 *SD* unit change in the predictor. Positive values indicate that curvature preferences increase as the trait increases.

#### Circles and hexagons

For the arrays of circles and hexagons, however, only one moderator was significant and another was marginal. As scores on the BFAS Intellect (*b* = −.15, *SE* = .08, *p* = .040) and the need for cognition (*b* = −.17, *SE* = .09, *p* = .068) scales increased, people tended to prefer the angular hexagons relatively more than the round circles. Otherwise, the effects for art expertise and openness to experience that appeared for the irregular polygons did not appear for the arrays (see Table 2).

### Does Curvature Affect Interest?

Our secondary aim was to investigate whether interest was influenced by curvature (see Figure 3). Angularity did not influence interest in the irregular polygon stimulus set (*b* = −.07, *SE* = .10, *p* = .463). Both the number of sides and range, however, affected interest. Shapes with more sides (*b* = .56, *SE* = .06, *p* < .001) and a higher range (*b* = .33, *SE* = .09, *p* < .001) were associated with increased interest ratings, consistent with a large body of work on complexity and interest (Silvia, 2006). The two-way interaction and the three-way interaction were not significant.

Similarly, in the circle and hexagons stimulus set, interest was not influenced by curvature (*b* = −.14, *SE* = .11, *p* = .21). The level of imbalance and the two-way interaction did not significantly affect interest ratings. In short, curved objects were pleasing, but angular objects did not appear to be more interesting.

### Did Curvature Affect Viewing Time?

Finally, we explored how curvature affected viewing time. As noted earlier, people could view each image as long as they wished with a minimum of 2 seconds. On average, people viewed the images for around 3.6 seconds (*M* = 3.57, *SD* = 1.66 for the irregular polygons; *M* = 3.65, *SD* = 1.90 for the circles and hexagons).

For the irregular polygons, there was a significant main effect of curvature (*b* = −.30, *SE* = .10, *p* = .002): People spent more time looking at the angular polygons than the rounded ones. There was also a main effect of the number of sides (*b* = .13, *SE* = .07, *p* = .048): Viewing time increased as the number of sides increased, a finding that replicates many studies from the Berlyne tradition of experimental aesthetics (e.g., Berlyne, 1974). Finally, there was a significant interaction between curvature and range (*b* = .30, *SE* = .14, *p* = .034): People spent more time viewing curved objects with a larger range.

For the arrays of circles and hexagons, there was no main effect of curvature on viewing time (*b* = −.06, *SE* = .12, *p* = .640). The only significant effect was a main effect of imbalance (*b* = −.14, *SE* = .05, *p* = .009): People spent more time viewing the more balanced arrays.

## Discussion

Among the many low-level features that affect aesthetic preference, curvature stands out as having an unusually strong evidence base. Preference for curved over angular forms has been demonstrated across decades, age-groups, cultures, and species (see Gómez-Puerto et al., 2015). When a main effect has been firmly established, a natural next step is to explore moderators. Marking the boundaries of an effect can illuminate its nature. Such illumination would be particularly welcome in the case of curvature preferences, which are robust yet mysterious—the effect is clearly there, but it is not obvious why adults, babies, and great apes would prefer the curved to the angular.

The present research examined individual differences in relation to curvature preferences. First, a preference for curved objects was found in both a set of irregular polygons and in arrays of circles and hexagons, replicating much past research. Second, we found evidence for individual differences in curvature preference itself. People’s liking for curved over angular shapes was consistent across the two stimulus sets, with a medium effect size. The consistency of people’s preferences across the types of shapes suggests the presence of individual differences that influence how much people prefer curved over angular forms.

The evidence for moderating traits, however, was a bit vexing. For one stimulus set—the irregular polygons generated and studied by Bertamini et al. (2016)—the evidence for moderation was plentiful and consistent. People with more knowledge about the arts, measured with the aesthetic fluency scale, showed greater curvature preferences, as did people higher in openness to experience, measured with the NEO, HEXACO, and BFAS. Thus, the participants who were more interested and knowledgeable showed a stronger relative preference for curved over angular polygons. Unlike other low-level factors that appear largely among novices, such as preferences for realistic over abstract images (Parsons, 1987), curvature preference was heightened among people with greater knowledge and interest in art, at least for this set of images. But for the other stimulus set—the arrays of circles and hexagons developed to study pictorial imbalance (Wilson & Chatterjee, 2005)—only a handful of effects appeared, and none were the same as in the set of irregular polygons.

Preference for curvature has been reported before for abstract stimuli (Bertamini et al., 2016) and for circles and hexagons (Silvia & Barona, 2009). Our study shows that beyond this similarity, the nature of the stimuli can be moderated differently by individual differences. For the irregular polygons, artistic expertise and openness to experience were significant predictors of preference for curvature. This was not visible for circles and hexagons. The irregular polygons were novel, whereas circles and hexagons are familiar objects. For instance, people possess linguistic labels for circles and hexagons, which come with a set of established semantic associations, such as between a circle and a ball, or between a hexagon and a beehive. It is likely that these stimuli already possess valence. It has been shown that valence can take precedence over curvature, and that in such cases, preferences for smooth curvature may be secondary (Leder et al., 2011). We conclude that there is a strong case for studying preference for shape properties using shapes that are novel and unfamiliar and that vary from trial to trial (i.e., they have a range of shapes, rather than just two).

Viewing time, along with pleasingness and interestingness, is one of the classic outcomes in experimental aesthetics research. To date, research on curvature has used a wide range of image exposures, such as under 100 ms (Bar & Neta, 2006; Munar et al., 2015) to 3 seconds (Vartanian et al., 2013), or has used free viewing without recording viewing times (e.g., Bertamini et al., 2016; Silvia & Barona, 2009, Study 2). In the present study, viewing time was influenced by some classic predictors, such as complexity and imbalance. In addition, for the irregular polygons, people spent more time viewing the angular shapes. The reason for this effect is unclear, but it does rule out a simple exposure explanation for the preference for curved objects. People overall preferred curved objects but did not spend more time viewing them, so preference is not merely due to exposure time.

Future work could vary presentation times to clarify why art expertise and openness to experience moderate people’s preference for curved objects. It is surprising that people with more expertise show greater curvature preferences because experts are often less affected by low-level aesthetic factors and more affected by formal training and domain knowledge (e.g., Haertel & Carbon, 2014; Parsons, 1987). It is possible that the free-viewing format allowed time for the effects of past experience and conceptual knowledge to materialize, so it would be worth varying presentation times and evaluating if traits like art expertise moderate preferences only at long viewing intervals. In addition, it would be worth greatly expanding the number of stimuli. We used relatively few in the current study to expand the time available for two tasks and many self-report questionnaires. Increasing the number of images could increase power for within-person effects.

A secondary question was whether curvature had different effects on liking and interest. It seemed possible that angular forms would seem more intriguing and interesting, given the large literature on how features associated with complexity, tension, and variety affect interest (Berlyne, 1971; Silvia, 2006). No evidence was found for effects on interest. The curved polygons and circle arrays were more pleasing, but the angular polygons and hexagon arrays were not any more or less interesting.

In sum, the present study found evidence for individual differences in curvature preferences. Some people showed stronger preference for curvature, and this preference was consistent for two very different types of shapes. The degree to which individual-difference factors moderated these preferences varied as a function of the type of shapes, suggesting that the familiarity of the shapes may be an important moderator of curvature preferences to examine in future research.

## Author Note

An early version of this study was presented at a symposium on “The Human Aesthetic (and Moral) Nature: The Preference for Curvature” at the University of the Balearic Islands. The stimulus materials and data are available at Open Science Framework (https://osf.io/vrjyp/).

## Note

1 Range was of secondary interest, but it did have some notable interactions. Range did not have a significant main effect on preference ratings (*b* = .02, *SE* = .08, *p* = .772). The two-way interaction between curvature and range, however, was significant (*b* = .44, *SE* = .12, *p* < .001): Participants liked the polygons more when both curvature and range were high. The two-way interactions between curvature and number of sides, and range and number of sides were not significant. A three-way interaction indicated that when curvature, number of sides, and range were high, participants liked the polygons less (*b* = −.24, *SE* = .08, *p* = .004).
